# Predictors of mortality in patients with severe sepsis or septic shock in the ICU of a public teaching hospital

**DOI:** 10.1186/cc12931

**Published:** 2013-11-05

**Authors:** Argenil José Assis de Oliveira, Cristina Padre Cardoso, Fabricio Rios Santos, Ana Paula de Magalhaes Campos, Edna Leite, Juliana de Assis Silva Gomes Stanislau, Fernando Antonio Botoni

**Affiliations:** 1 Postgraduate Course of Tropical Medicine, Federal University of Minas Gerais, Belo Horizonte, MG, Brazil; 2 Hospital Risoleta Tolentino Neves, Federal University of Minas Gerais, Belo Horizonte, MG, Brazil; 3 Institute of Biological Sciences, Federal University of Minas Gerais, Belo Horizonte, MG, Brazil; 4 Universidade Federal do Mato Grosso, Brazil; 5 Hospital Infection Control Center, Hospital Risoleta Tolentino Neves - Federal University of Minas Gerais, Belo Horizonte, MG, Brazil; 6 Department of Internal Medicine, School of Medicine, Federal University of Minas Gerais, Belo Horizonte, MG, Brazil

## Background

Sepsis is a complex and multifactorial syndrome, whose incidence, morbidity and mortality have been increasing worldwide. The knowledge of clinical, epidemiological and hemodynamic parameters responsible for its evolution, diagnosis and treatment are still the subject of many studies. Therefore, this study aims to evaluate clinical, laboratory and hemodynamic parameters of morbidity and mortality in patients with severe sepsis and septic shock.

## Materials and methods

As the diagnostic criteria of the systemic inflammatory response syndrome (SIRS) are very sensitive and very little specific, we selected patients with severe sepsis and septic shock in the first 24 hours of ICU admission, 18 years old or more, with two general and one or more inflammatory criteria of SIRS (ACCP/SCCM/2003). Patients with pathologies that could confound clinical and laboratory evaluations and advanced comorbidities or on immunosuppressive drug therapy were excluded. The ICU had 35 beds, five of them are resuscitation beds located in the emergency room (ER). The same intensivist team assists all patients in the ER and during ICU permanence. The principal investigator did not perform any orientation or intervention in the treatment of selected patients. Clinical (age, sex, infection focus, fluid balance, hemodialysis, use of corticosteroids, antibiotic therapy, APACHE II, SOFA), laboratory (blood cell counting, lactate, creatinine, bilirubin, glucose, cortisol, NT-proBNP, C-reactive protein (CRP), procalcitonin (PCT), Troponin I), hemodynamic (blood pressure, heart rate, left ventricular systolic function (echocardiography)) and respiratory parameters (respiratory rate, PaO_2_/FiO_2_), PEEP and peak inspiratory pressure (PIP)) were analyzed from ICU admission until discharge or death. Echocardiography was performed at 48 hours and on the 10th day after ICU admission.

## Results

Seventy-two patients (64% male), mean age 52 ± 19 years, were consecutively included, 21% (15/72) with severe sepsis and 79% (57/72) with septic shock. Mortality was 18% (13/72), of these 21% (3/13) for severe sepsis and 79% (10/13) for septic shock. Median APACHE II score was 28 (16 to 37) and SOFA score 6 (5 to 10) (Table 1). There was positive correlation between mortality with: male gender, APACHE II, SOFA, positive 24-hour fluid balance, hemodialysis indication, corticosteroid use, leukopenia, lactate, NT-proBNP and PCT levels (Table 2). From univariate analysis, practically the same significant association with mortality was observed (Table 3). In addition, the final multivariate Cox model showed that male gender, hypotension (first 24 hours), leukopenia and positive fluid balance (first 24 hours) had an impact on mortality (Table 4). Glycemic control and early antibiotic use were not relevant.

**Table 1:** Demographic and clinical characteristics observed in patients with severe sepsis and septic shock

Variable	*n *= 72
Age, median (years; IQR)	53 ± 19
Male gender	64%
Type of admission	
Medical	33 (47%)
Surgical	39 (53%)
ICU admission source	
Emergency room	34 (47%)
Surgical room	38 (53%)
Interval between hospital admission and ICU	
>24 hours	20 (28%)
<24 hours	52 (72%)
Physical examination	
Peripheral edema	46 (63.9%)
Capillary refilling time reduced	45 (62.5%)
Sedation	
Yes	55 (76.4%)
No	17 (23.6%)
Comorbidities	
Hypertension	23 (31.9%)
Diabetes	11 (15.3%)
Alcoholism	30 (41.6%)
Smoking	29 (40%)
Chronic renal failure	7 (9.7%)
Hemodialysis	18 (25%)
Blood products	39 (54.2%)
Cardiac arrest rate during ICU stay	4 (2.88%)
APACHE II, median (IQR)	28 (18 to 34)
SOFA score	
Initial, median (IQR)	3 (2 to 8)
Media, median (IQR)	6 (5 to 10)
Maximum, median (IQR)	11 (7 to 13)
Severe sepsis	15 (20.8%)
Septic shock	57 (79.2%)
ICU stay, median (days; IQR)	8 (4 to 15)
Hospital stay, median (days; IQR)	20 (8 to 40)
Positive cultures	
Blood	12 (17%)
Sterile tissue or cavity	11 (15%)
ICU mortality	13 (18%)
Hospital mortality	13 (18%)

Demographic and clinical characteristics observed in patients with severe sepsis and septic shock in the ICU/HRTN between April 2011 and October 2012. IQR, interquartile range.

**Table 2:** Clinical and laboratory variables related to mortality in patients with severe sepsis and septic shock

Variable	Correlation *r*	*P *value
Male gender	0.248	0.05
Age	0.309	0.01
APACHE II score	0.478	0.01
SOFA score	0.572	0.01
Fluid balance (24 hours)	0.350	0.01
Fluid balance (7 days)	0.590	0.01
Hemodialysis	0.548	0.01
Tachycardia	0.266	0.01
PaO_2_/FiO_2_	0.320	0.01
Vasopressor agent (24 hours)	0.445	0.01
MAP (24 hours)	0.485	0.01
Creatinine	0.548	0.01
Lactate	0.375	0.01
Steroid use	0.337	0.01
Leukopenia	0.404	0.01
NT-pro-BNP	0.269	0.05
PCT	0.320	0.01

Clinical and laboratory variables related to the mortality observed in patients with severe sepsis and septic shock in the ICU/HRTN between April 2011 and October 2012 (*n *= 72). Data expressed as Spearman *r *and *P *value. MAP, mean arterial pressure; NT-pro-BNP, N-terminal natriuretic peptide; PCT, procalcitonin.

**Table 3:** Univariate analysis of variables associated with mortality in patients with severe sepsis and septic shock

Variable	HR	95% CI	*P *value
Male gender	3.24	1.13 to 22.40	0.06
Age	1.05	0.92 to 13.70	0.00
MAP 24 hours	0.79	0.71 to 0.90	0.00
Tachycardia	1.03	1.00 to 1.06	0.03
Fluid balance (24 hours)	1.00	1.00 to 1.00	0.00
Vasopressor use (24 hours)	1.18	0.84 to 2.80	0.00
Steroid use	5.28	2.30 to 11.80	0.00
Leukopenia	5.36	0.97 to 29.6	0.05
Mechanical ventilation (*t*)	0.762	0.60 to 0.96	0.12
PEEP	1.24	0.70 to 1.70	0.08
PIP	1.22	1.07 to 1.39	0.04
Hemodialysis	6.29	2.05 to 19.2	0.00
PCT	1.14	0.90 to 1.30	0.06
Lactate	9.55	1.12 to 73.96	0.03
Creatinine	4.07	0.99 to 22.32	0.01
APACHE II score	1.09	0.977 to 1.23	0.00
SOFA score	1.24	1.06 to 1.44	0.00

Univariate analysis of variables associated with mortality observed in patients with severe sepsis and septic shock in the ICU/HRTN between April 2011 and October 2012 (*n *= 72). MAP, mean arterial pressure; PCT, procalcitonin; PEEP, positive end expiratory pressure; PIP, peak inspiratory pressure; *t*, time (days).

**Table 4:** Multivariate analysis of variables associated with mortality in patients with severe sepsis and septic shock

Variable	HR	95% CI	*P *value
MAP (24 hours)	0.74	0.64 to 0.85	0.0001
Fluid balance (24 hours)	1.001	1.000 to 1.001	0.002
Male gender	5.35	1.10 to 26.15	0.038
Leukopenia	60.85	4.97 to 74	0.001

Multivariate analysis of variables associated with mortality observed in patients with severe sepsis and septic shock in the ICU/HRTN between April 2011 and October 2012 (*n *= 72). CI, confidence interval; HR, hazard ratio; MAP, mean arterial pressure.

## Conclusions

Precocious treatment, judicious fluid management and individualized care showed benefit in the treatment of patients with severe sepsis, septic shock.

